# Prenatal Valproic Acid Exposure Impairs Offspring Cognition Through Disturbing Interneuron Development

**DOI:** 10.1111/cns.70303

**Published:** 2025-02-27

**Authors:** Kaiyuan Shen, Yandong Zhang, Yunyun Huang, Yunli Xie, Jing Ding, Xin Wang

**Affiliations:** ^1^ Department of Neurology Zhongshan Hospital, Fudan University Shanghai China; ^2^ State Key Laboratory of Medical Neurobiology and MOE Frontiers Center for Brain Science, Institutes of Brain Science Fudan University Shanghai China

**Keywords:** antiseizure medication, cognition, neurodevelopment, pregnancy with epilepsy

## Abstract

**Aims:**

Valproic acid (VPA) exposure during the gestational period has been found to impair the cognition of the offspring. The study aimed to investigate whether VPA leads to offspring cognitive impairment through disturbing interneuron development.

**Methods:**

Pregnant mice were injected with VPA peritoneally to establish the prenatal VPA exposure model. Cortical interneurons were labeled with Rosa26‐EYFP/− reporter mice activated by Nkx2.1‐Cre. Interneuron subtypes both in the cortex and the hippocampus were detected by immunofluorescence. A battery of behavioral tests was conducted on postnatal Day 28 to assess the cognition and anxiety of the offspring. RNA‐Seq analysis was performed to investigate the underlying molecular mechanisms.

**Results:**

We found that after the exposure to VPA, all the groups of the male offspring exerted anxiety. When VPA injection was performed on gestational Day 12.5, the memory of the offspring was impaired. Mechanistically, the distribution of cortical interneurons was disrupted. The distribution of interneuron subtypes was abnormal both in the cortex and hippocampus after the VPA exposure, which affected the somatostatin‐positive neurons but not the parvalbumin‐positive neurons, indicating the effects of VPA were subtype specific. Biological processes related to ion homeostasis were greatly changed after VPA exposure.

**Conclusion:**

Prenatal VPA exposure during the neurogenic period impaired the cognition of the offspring by disrupting interneuron migration and differentiation. The study provides a novel perspective on the influence of VPA over neurodevelopment.

## Graphical abstract

Prenatal VPA exposure during the neurogenic period impaired the cognition of the offspring by disrupting interneuron migration and differentiation. The influence of VPA was found to be subtype‐specific, predominantly affecting the somatostatin‐positive neurons.

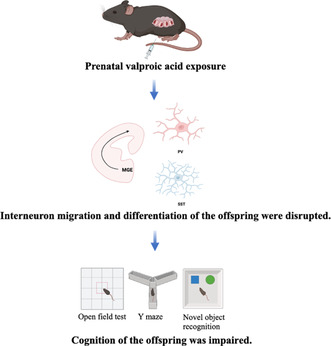

## 1 Introduction

Epilepsy is a common neurological disorder affecting over 70 million people worldwide, with an incidence rate of 40–70 per 100,000 person‐years [1]. The prevalence among women is around 6.85‰, while in pregnant women it stands at 0.3%–0.7% [2]. Antiseizure medications (ASMs) are the most commonly applied treatment to control seizures. However, many ASMs are currently considered teratogenic [3]. The incidence of major congenital abnormalities in offspring increased by 1.37–3.04 times [4] after taking ASMs such as valproic acid (VPA), ethosuximide, topiramate, phenobarbital, phenytoin sodium, and carbamazepine.

Valproic acid (VPA) is a broad‐spectrum ASM used to treat various types of epilepsy, as well as to alleviate migraines and psychiatric disorders. Research indicates that VPA impedes neurodevelopment and causes cognitive impairments in offspring, leading to conditions such as autism spectrum disorder and attention deficit hyperactivity disorder [5, 6]. Retrospective studies have observed cognitive decline in children with intrauterine VPA exposure at ages 3 [7], 6 [8] and 10–12 years [9]. The Food and Drug Administration (FDA) has issued a warning about the risk of cognitive impairment in offspring when VPA is taken during pregnancy [10]. Despite the warning, VPA remains widely used during pregnancy for controlling specific types of epilepsy and in areas with limited access to alternative medications. Understanding the mechanisms by which VPA affects neurodevelopment in offspring is crucial for optimizing treatment and seeking potential solutions.

Interneurons (INs) are widely distributed γ‐aminobutyric acid (GABA)‐ergic neurons in the brain, accounting for about 20% of cortical neurons [11]. Cortical interneurons generate from the proliferative regions around the lateral ventricles of the embryonic brain, including the medial ganglionic eminence (MGE), caudal ganglionic eminence (CGE) and preoptic area (POA) [12]. Interneurons expressing parvalbumin (PV) and somatostatin (SST), which constitute 70% of cortical interneurons, derive from the MGE [13]. In mice, the genesis of cortical interneurons begins at embryonic day (E) 11.5, involving processes such as neurogenesis, migration, programmed cell death, and circuit formation [14]. This development is precisely regulated by both intrinsic and extrinsic factors [15]. Environmental influences, such as drug usage and infections during pregnancy, can disrupt the development of interneurons [16, 17]. Therefore, VPA exposure as an extrinsic factor has the potential to interfere with the process.

Interneurons play a crucial role in preventing overexcitation and synchronizing the electrical activity of excitatory neurons, which is essential for the formation of cortical circuits and maintaining excitatory/inhibitory (E/I) balance in the brain [18]. E/I balance is fundamental to cortical function. E/I imbalance can lead to developmental disorders and cognitive impairment [19]. Mutations in genes encoding transcription factors that regulate interneuron development, such as CHD2 [20], FOXP1 [21] and TCF4 [22], have been linked to conditions like epilepsy, autism spectrum disorder (ASD) and schizophrenia.

In this study, we investigated the negative impact of VPA exposure on offspring cognition. We hypothesized that prenatal VPA exposure impaired the cognition of the offspring by disturbing interneuron development.

## 2 Materials and Methods

### 2.1 Animals

All experimental procedures were performed in accordance with guidelines approved by the Animal Care and Use Committee of Fudan University. Mice were maintained in accordance with institutional guidelines. Six‐week‐old C57BL/6 mice were purchased from the SLAC Laboratory. Transgenic heterozygous Rosa26‐EYFP/− mice and Nkx2.1‐Cre/− mice were previously described [23]. Mice were housed at a constant temperature of 23°C–24°C, with a 12‐h light/dark cycle, and had free access to food and water. Two female mice and one male mouse were housed per cage. The presence of vaginal plugs was recorded as embryonic day (E) 0.5.

### 2.2 VPA Exposure

Pregnant mice were administered 300 mg/kg of VPA via intraperitoneal injection. They were randomly assigned to one of the following groups: the control (Ctr) group, which received 200 μL of 0.9% saline solution; the E10.5 group, which received VPA at E10.5; the E12.5 group, which received VPA at E12.5; or the E14.5 group, which received VPA at E14.5.

### 2.3 Behavioral Tests

The behavioral tests were performed following the published protocol [24]. To minimize the influence of hormonal changes on the behavioral experiments, only male offspring mice were included in the study.

#### 2.3.1 Open Field Test (OFT)

Male offspring mice at P28 were given 30 min to acclimate to the environment before the test began. Test equipment was cleaned with 75% alcohol. Mice were gently placed in the center of the open field. Enthovision software automatically recorded their activity for 10 min. After the test, the mice were returned to their cages. Both the time spent and the distance traveled by the mice were recorded.

#### 2.3.2 Novel Object Recognition Test (NORT)

Following the OFT, the novel object recognition test (NORT) was conducted. Two toy bricks, tall enough to prevent the mice from jumping onto them, were placed in the equipment. The bricks were secured to ensure that the mice could not move them. Enthovision software recorded instances of the mice exploring the objects when their noses were within 2 cm of the bricks. Each mouse was given 10 min to familiarize itself with the bricks. The equipment was then cleaned with 75% alcohol. The next day, one of the bricks was replaced with a new brick. Mice were reintroduced into the field for 10 min to explore the bricks, and the exploration was recorded by the software.

#### 2.3.3 Y Maze Test

Y maze test was conducted 3 days after NORT. Mice were placed in the center of a Y‐shaped maze with three arms. As soon as the mice entered the maze, Enthovision software began monitoring their movements. Entries into the arms were recorded over a 10‐min period. The environment was maintained quiet to avoid unnecessary interference.

### 2.4 Immunofluorescence Staining

Brains were perfused with a 0.9% saline solution and then fixed overnight at 4°C with 4% paraformaldehyde (PFA). Subsequently, the brains were transferred to a 30% sucrose solution for 24 h and embedded in the OCT compound (Sakura). Postnatal brains were sectioned at a thickness of 30 μm. The brain slices were washed with phosphate‐buffered saline (PBS) for 30 min and permeabilized with 0.5% Triton X‐100 in PBS for another 30 min at room temperature. After permeabilization, the slices were incubated in a blocking solution (0.3% Triton X‐100, 5% normal donkey serum in PBS) for 30 min. The slices were then incubated with primary antibodies overnight at 4°C. The following day, the slices were washed again with PBS for 30 min and subsequently incubated with secondary antibodies and DAPI (0.5 μg/mL in PBS, Sigma) in a dark room for 2 h.

### 2.5 RNA‐Seq and Analysis

Cerebral cortex at P0 was isolated from the Ctr group and VPA group. RNA‐Seq was performed at Shanghai Genergy Bio‐technology Co. The data were analyzed using DESeq2 and TopGO software. Differential expression was performed with a cut‐off of FDR < 0.05 and abs (log_2_ FC) > 1.0. The volcano plots and the heatmap were generated by R language.

### 2.6 Image Acquisition and Analysis

At least five mice from different mothers were analyzed in each group. Images were captured using a fluorescence microscope (Nikon) with a 10X objective. The images were processed using NIS‐Elements AR (Nikon) and ImageJ software.

### 2.7 Statistical Analysis

GraphPad Prism 10 was utilized for data analysis and graphical presentation. For analyzing more than two groups, one‐way ANOVAs were conducted, while two‐way ANOVAs and Bonferroni's post hoc tests were performed for two parameters across multiple groups. All data were presented as mean ± standard error of the mean (SEM). A *p*‐value of less than 0.05 was considered statistically significant. Asterisks indicated the level of statistical significance (*p < 0.05; **p < 0.01; ***p < 0.001).

## 3 Results

### 3.1 VPA Exposure Did Not Cause Major Developmental Abnormalities in Offspring

**FIGURE 1:**
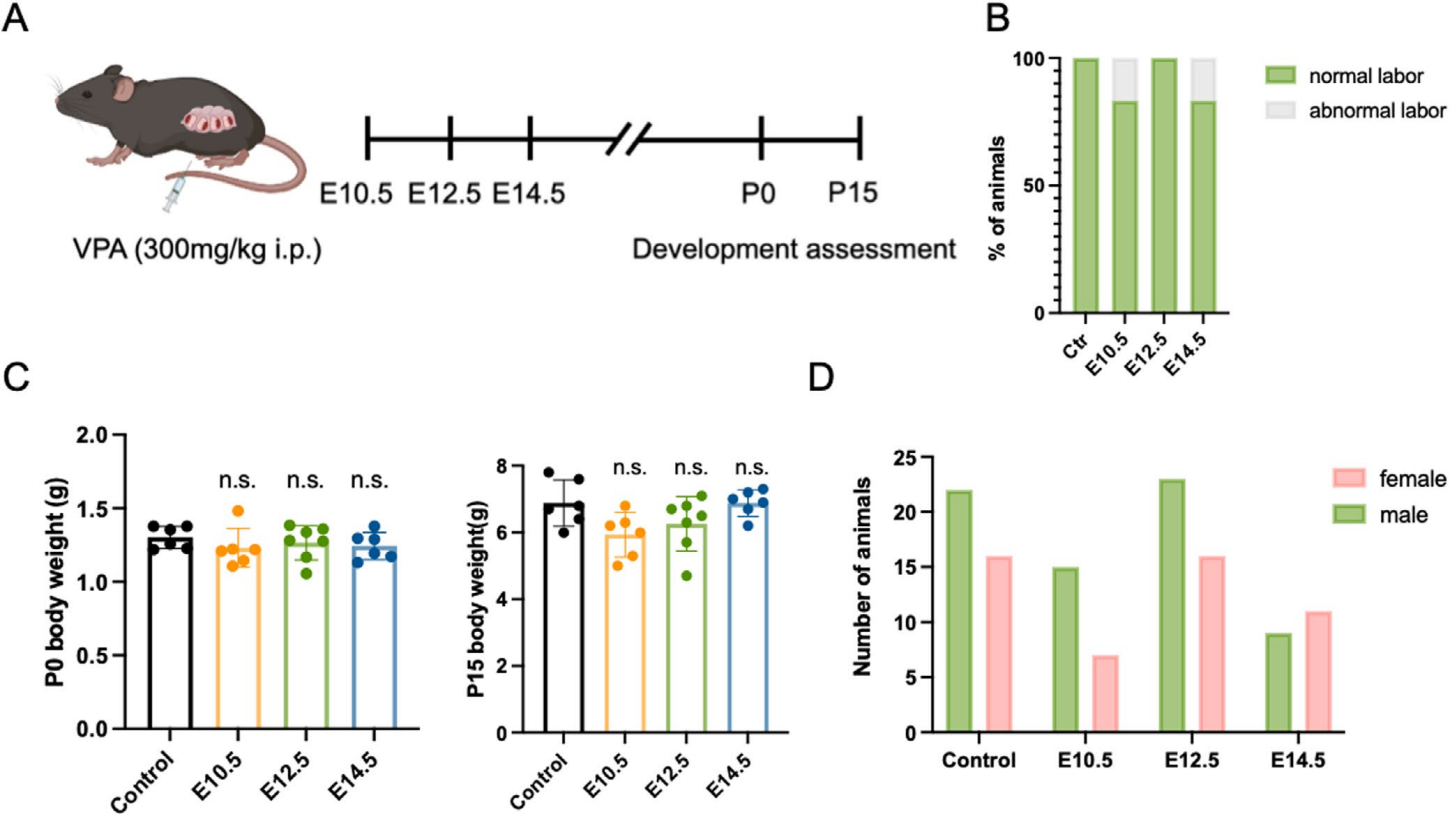
Prenatal valproic acid exposure did not cause major development abnormalities in offspring. (A) Experimental schedule. (B) Quantification of the normal labor rate and abnormal labor rate in the four groups. (C) Offspring body weights of the four groups at postnatal Day 0 and postnatal Day15. (D) Number of the offspring mice in four groups. All bar plots show mean ± SEM. Ctr: *N* = 6; E10.5: *N* = 6; E12.5: *N* = 7; E14.5: *N* = 6. One‐way ANOVA was performed in (C). Chi‐square test was performed in (D). n.s. no significance, as indicated.

We established a non‐teratogenic mouse model to focus on investigating the effects of prenatal valproic acid (VPA) exposure on the development of offspring brains. Major organs, such as the heart, intestine, and neural crest generate before embryonic day10.5 (E10.5). Therefore, we administered VPA after E10.5. Pregnant mice were randomly assigned to be exposed to normal saline (Ctr), VPA at E10.5, VPA at E12.5, and VPA at E14.5 (Figure 1A) to determine whether the effects of VPA on neurodevelopment are time‐dependent. The anti‐seizure dose of VPA in mouse models ranges from 200 to 570 mg/kg [25, 26]. To evaluate the effects of VPA at therapeutic doses, we administered an intraperitoneal injection of 300 mg/kg to the pregnant mice. In this study, pregnant mice that failed to give birth on time at E19.5 or whose offspring died before postnatal Day 2 (P2) were identified as having abnormal labor. The abnormal labor rate was 0% in both the Ctr and E12.5 groups, while it was 16.67% in the E10.5 and E14.5 groups (Figure 1B). We compared the body weights of offspring at P0 and P15 across all groups and found no significant differences (Figure 1C). We also recorded the gender of the offspring, and the gender ratio was similar among the four groups (Figure 1D). These results showed that the use of valproic acid during pregnancy in this study did not cause major developmental abnormalities in the offspring mice.

### 3.2 Prenatal VPA Exposure Impaired the Cognition of Adolescent Offspring

**FIGURE 2:**
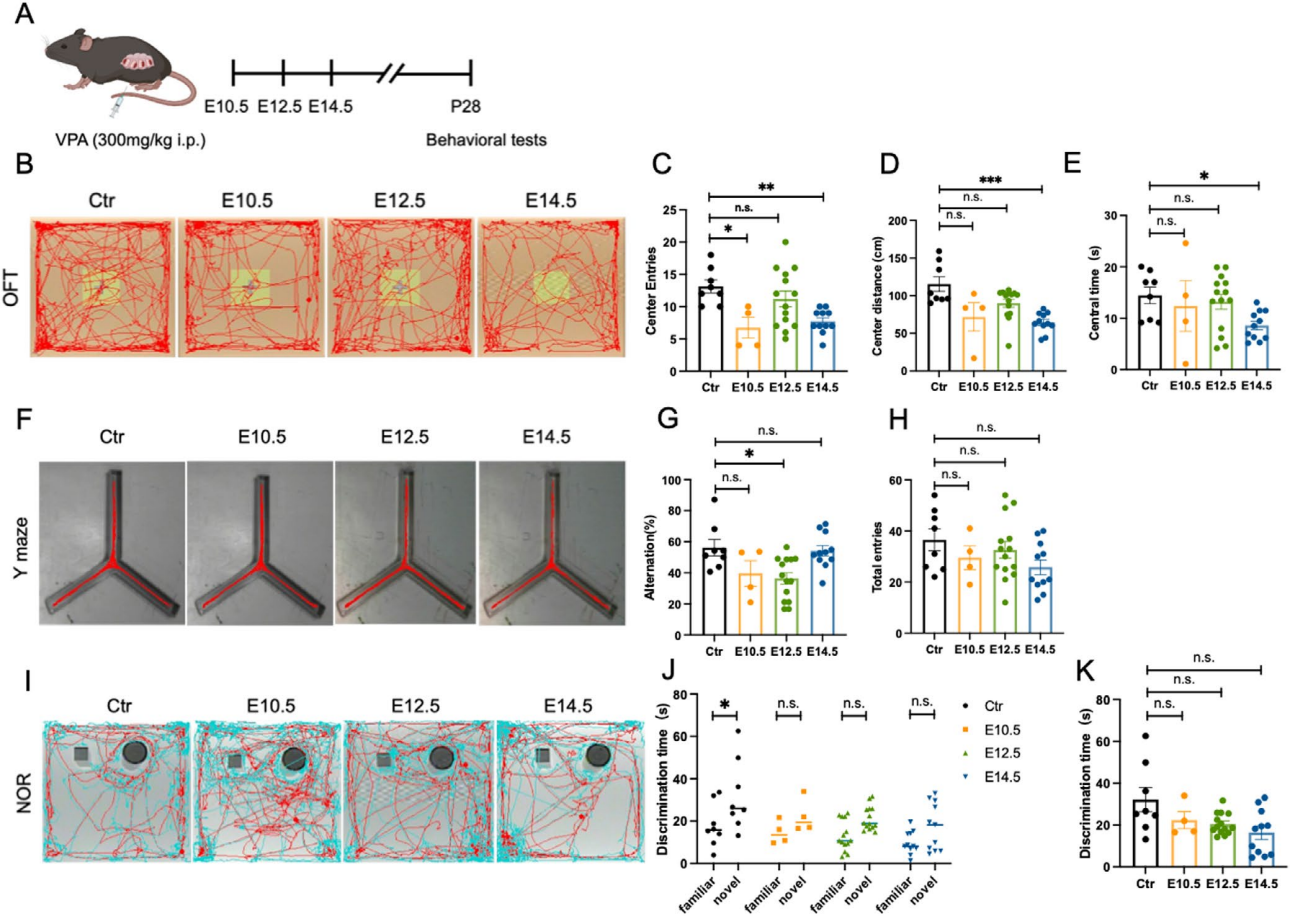
Prenatal valproic acid exposure impaired the cognitive behavior of the adolescent offspring. (A) Experimental schedule. (B) Representative traces in the OFT were displayed. OFT was used to determine whether the offspring were anxious(C–E). (C) Quantification of the numbers mice entered the center. (D) Quantification of the distance mice traveled in the center. (E) Quantification of the time mice spent in the center. Y maze test was used to detect the working memory of the offspring (F–H). (F) Representative traces in the Y maze were displayed. (G) Quantification of the alternation rate. (H) Quantification of the total arm entry numbers. NOR was used the test the short‐term memory of the offspring (I–K). (I) Representative traces in the NOR were displayed. (J) Quantification of the time spent with the familiar or novel object. (K) Quantification of the time spent with the novel object. All bar plots show mean ± SEM. Ctr: *N* = 8; E10.5: *N* = 4; E12.5: *N* = 14; E14.5: *N* = 11. Kruskal–Wallis test was performed in (C–E, G, H, K). Two‐way ANOVA was performed in (J). ***p < 0.001, **p < 0.01, and *p < 0.05, ns no significance, as indicated.

Retrospective studies have shown that children with intrauterine exposure to VPA suffer from cognitive disorders at ages 3, 6, and 10–12 years [7, 8, 9]. To examine the effects of VPA on cognition, behavioral tests were conducted on male offspring mice. The open field test was used to assess anxiety. Offspring exposed to VPA at E10.5 and E14.5 entered the center less frequently and traveled a shorter distance compared to the Ctr group (Figure 2C–E). To evaluate spatial memory, we performed the Y‐maze test. Normally, mice tended to enter alternative arms of the Y maze. However, the alteration rate declined in the E12.5 group, indicating an impairment in spatial memory (Figure 2G). The total number of arm entries was similar across all groups (Figure 2G,H), suggesting that motor function was not significantly affected. The novel object recognition test was used to test long‐term memory, as mice had an instinct to explore new objects. Only the offspring in the Ctr group showed a tendency to explore new objects (Figure 2J,K), indicating impaired long‐term memory in these groups.

### 3.3 Prenatal VPA Exposure Altered the Migration of Cortical Interneurons

**FIGURE 3:**
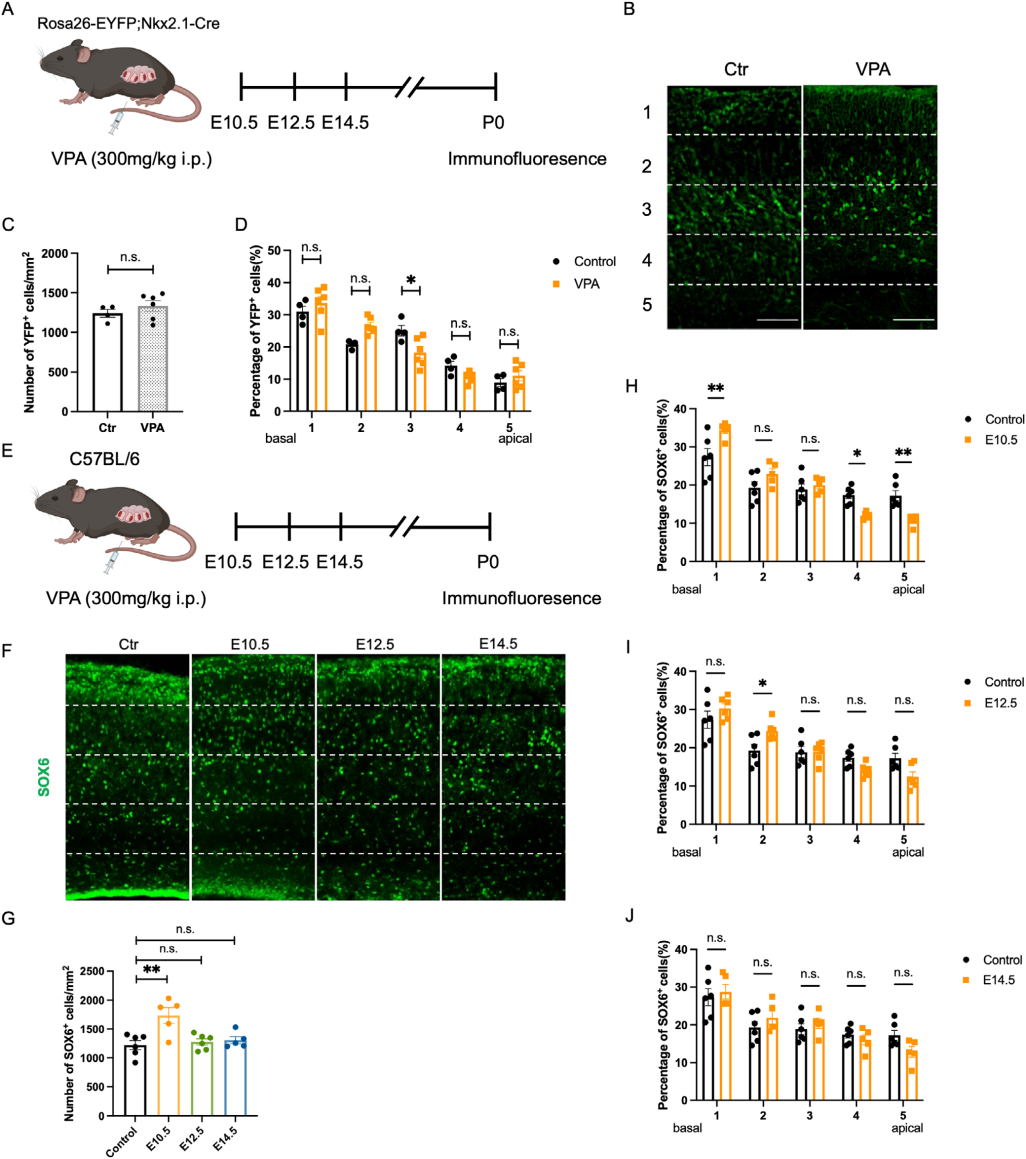
Prenatal VPA exposure disturbed the interneuron migration in the postnatal prefrontal cortex of the offspring. (A) Experimental schedule. (B) Representative images of YFP^+^ immunofluorescence staining in the prefrontal cortex of the offspring at P0. Scale bar = 100 μm. (C) Quantification of YFP^+^ cells in the prefrontal cortex at P0 of mice with maternal VPA exposure at E10.5, E12.5 and E14.5. (D) Cortical sections of the mice with maternal VPA exposure at E10.5, E12.5 and E14.5 were divided into 5 equal‐sized bins and the number of YFP^+^ cells were counted in each. Bin 1 and Bin 5 represent the basal and apical side of the prefrontal cortex respectively. The number of YFP^+^ cells in different bins at P0 were analyzed. Ctr: *N* = 4;VPA: *N* = 6. (E) Experimental schedule. (F) Representative images of SOX6^+^ immunofluorescence staining in the prefrontal cortex of the offspring at P0. Scale bar = 100 μm. G. Quantification of SOX6^+^ cells in the prefrontal cortex at P0. The number of SOX6^+^ cells in different bins at P0 after the treatment of VPA at E10.5 (G), E12.5 (H) and E14.5 (I) were analyzed. Cortical sections were divided into 5 equal‐sized bins and the number of SOX6^+^ cells were counted in each (H–J). Bin 1 and Bin 5 represent the basal and apical side of the prefrontal cortex respectively. Ctr: *N* = 6; E10.5: *N* = 5; E12.5: *N* = 6; E14.5: *N* = 5. All bar plots show mean ± SEM. One‐way ANOVA were performed in (C, G). Two‐way ANOVA were performed in (D, H–J). **p < 0.01, and *p < 0.05, ns no significance, as indicated.

Interneurons play a crucial role in maintaining the cortical E/I balance, which is vital for cognitive function. Previous studies reported an abnormal distribution of cortical neurons in offspring mice exposed to VPA, but the specific types of neurons were yet to be clarified [27]. We attempted to figure out whether cortical interneurons were involved. In rodents, most of interneurons (~70%) derive from MGE and migrate to the cortex. Nkx2.1 is a key regulator of the MGE‐derived interneurons. To detect the distribution of interneurons, we collected brains from Rosa26‐EYFP/−; Nkx2.1‐Cre mice at P0 (Figure 3A). Pregnant mice were injected with 300 mg/kg VPA intraperitoneally at E10.5, E12.5, and E14.5. Immunostaining suggested that VPA exposure during pregnancy did not alter the total number of cortical interneurons in the offspring (Figure 3C). However, the distribution of cortical interneurons was changed (Figure 3D). Further, we examined the effects of VPA on cortical interneurons at different time points. SOX6, which is present in most MGE neurons and plays an important role in interneuron maturation and positioning, was used as a marker for interneurons (Figure 3F). Results showed an increase in the number of SOX6^+^ cells in the cortex of mice exposed to VPA at E10.5 (Figure 3G). The distribution of cortical interneurons was disarranged in both the E10.5 and E12.5 groups (Figure 3H,I). These findings indicated that prenatal VPA exposure might impair interneuron migration.

### 3.4 Prenatal VPA Exposure Disturbed the Distribution of Cortical Somatostatin Interneurons

**FIGURE 4:**
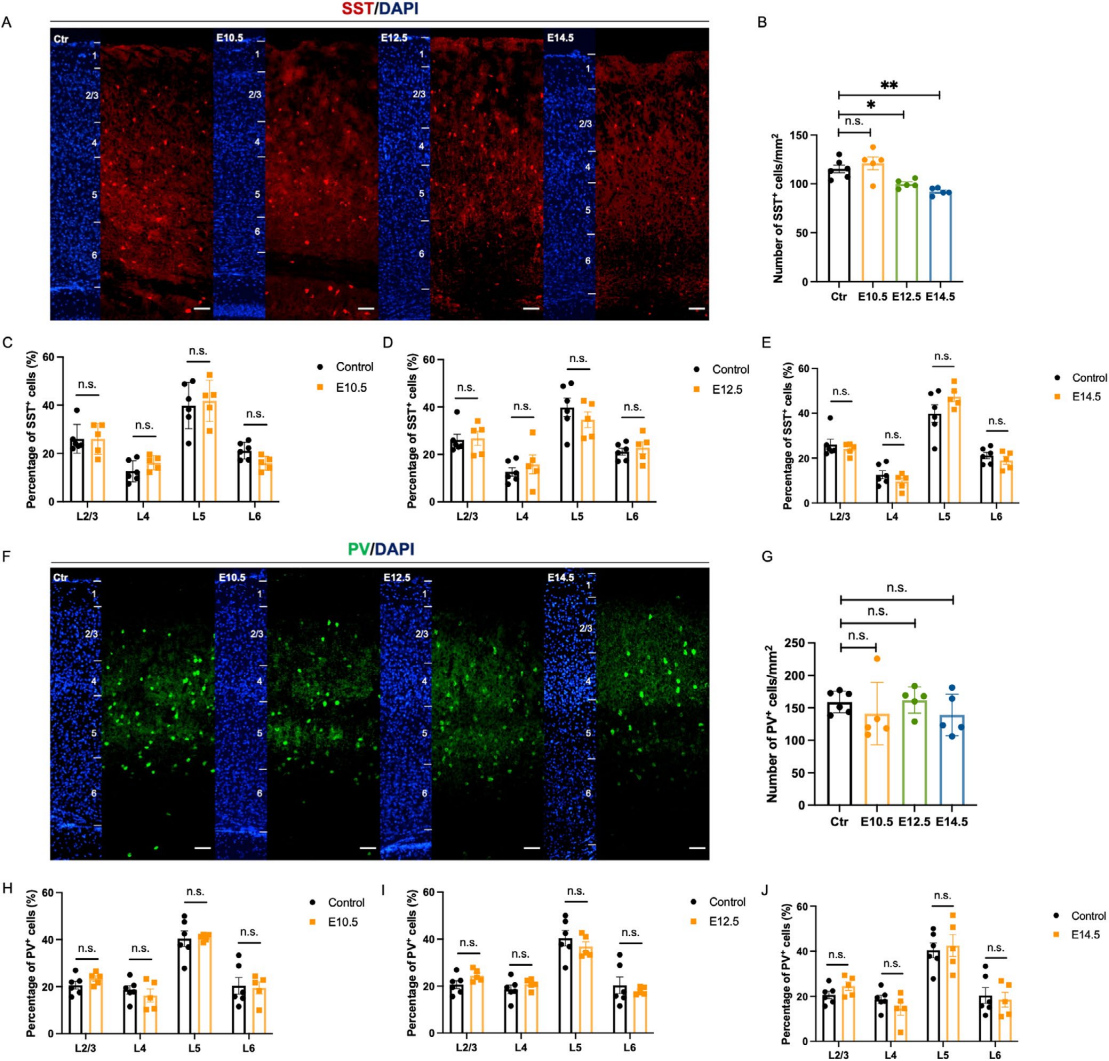
Prenatal VPA exposure impaired the distribution of the offspring cortical somatostatin interneurons. (A) Representative images of SST^+^ immunofluorescence staining in the prefrontal cortex of the offspring at P42. Scale bar = 100 μm. (B) Quantification of SST^+^ cells in the prefrontal cortex at P42. (C–E) Percentages of SST^+^ cells in the different layers of the cortex at P42 after the treatment with VPA at E10.5 (C), E12.5 (D) and E14.5 (E). Ctr: *N* = 6; E10.5: *N* = 5; E12.5: *N* = 5; E14.5: *N* = 5. (F) Representative images of PV^+^ immunofluorescence staining in the prefrontal cortex of the offspring at P42. Scale bar = 100 μm. (G) Quantification of PV^+^ cells in the prefrontal cortex at P42. (H–J) Percentages of PV^+^ cells in the different layers of the cortex at P42 after the treatment with VPA at E10.5 (H), E12.5 (I) and E14.5 (J). Ctr: *N* = 6; E10.5: *N* = 5; E12.5: *N* = 5; E14.5: *N* = 5. All bar plots show mean ± SEM. One‐way ANOVA were performed in (B, G). Two‐way ANOVA were performed in (C–E, H–J). **p < 0.01, and *p < 0.05, ns no significance, as indicated.

Abnormal localization of interneurons is also associated with neurodevelopmental diseases. Interneurons are highly heterogeneous and can be classified into various subtypes based on their morphological and electrophysiological characteristics. Among them, PV^+^ and SST^+^ neurons account for the majority. To investigate the effects of VPA on interneuron distribution, we examined PV^+^ and SST^+^ cells in the cortex of the offspring mice at P42 by immunofluorescence (Figure 4A,F). The number of cortical SST^+^ cells decreased in the E12.5 and E14.5 groups (Figure 4B). However, the proportion of SST^+^ cells in each layer are similar (Figure 4C–E). There were no significant changes in the number and distribution of PV^+^ cells in the cortex of the offspring (Figure 4G–J). The results suggested that prenatal VPA exposure interfered with the distribution of cortical interneurons, with a primary impact on the SST^+^ subtype, indicating that the effects of VPA might be subtype‐specific.

### 3.5 Prenatal VPA Exposure Disturbed the Distribution of Hippocampal Somatostatin Interneurons

**FIGURE 5:**
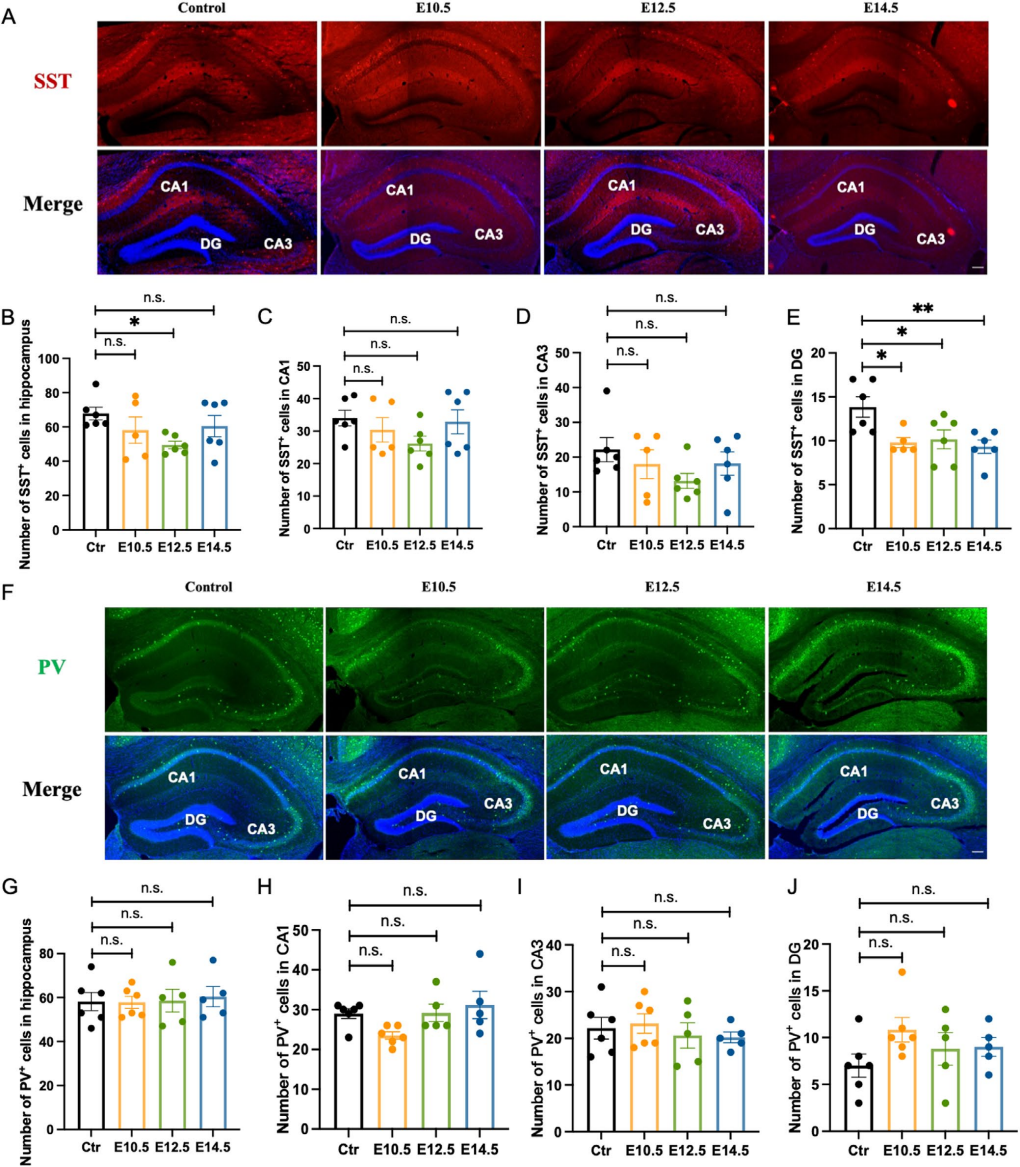
Prenatal VPA exposure impaired the distribution of the offspring hippocampal somatostatin interneurons. (A) Representative images of SST^+^ immunofluorescence staining in the hippocampus of the male offspring at P42. Scale bar = 200 μm. (B) Quantification of the total SST^+^ cells in the hippocampus at P42. Quantification of SST^+^ cells in the CA1 (C), CA3 (D) and DG (E) region of the hippocampus at P42. Ctr: *N* = 6; E10.5: *N* = 5; E12.5: *N* = 6; E14.5: *N* = 6. (F) Representative images of PV^+^ immunofluorescence staining in the hippocampus of the offspring at P42. Scale bar = 200 μm. (G) Quantification of the total PV^+^ cells in the hippocampus at P42. Quantification of PV^+^ cells in the CA1 (H), CA3 (I) and DG (J) region of the hippocampus at P42. Ctr: *N* = 6; E10.5: *N* = 6; E12.5: *N* = 5; E14.5: *N* = 5. All bar plots show mean ± SEM. One‐way ANOVA were performed in (B–E, G–J). **p < 0.01, and *p < 0.05, ns no significance, as indicated.

Hippocampus is vital for cognitive functions such as memory. MGE progenitors also produce PV^+^ and SST^+^ cells that migrate to the hippocampus, making up about 60% of total hippocampal interneurons [28]. Therefore, we further investigated the development of hippocampal interneurons in the offspring mice at P42 (Figure 5A,F). The total number of SST^+^ cells in the hippocampus decreased in the E12.5 group (Figure 5B). Additionally, the number of SST^+^ cells in the dentate gyrus (DG) declined in all groups exposed to VPA during pregnancy (Figure 5E). However, the number of PV^+^ cells in the entire hippocampus, as well as in CA1, CA2, and DG regions, remained unchanged (Figure 5G–5J). Similar to the findings in the cortex, the results showed that prenatal VPA exposure had subtype‐specific effects in the hippocampus, mainly affecting SST^+^ cells.

### 3.6 Valproic Acid Exposure Altered Gene Expressions Related to Interneuron Development

**FIGURE 6:**
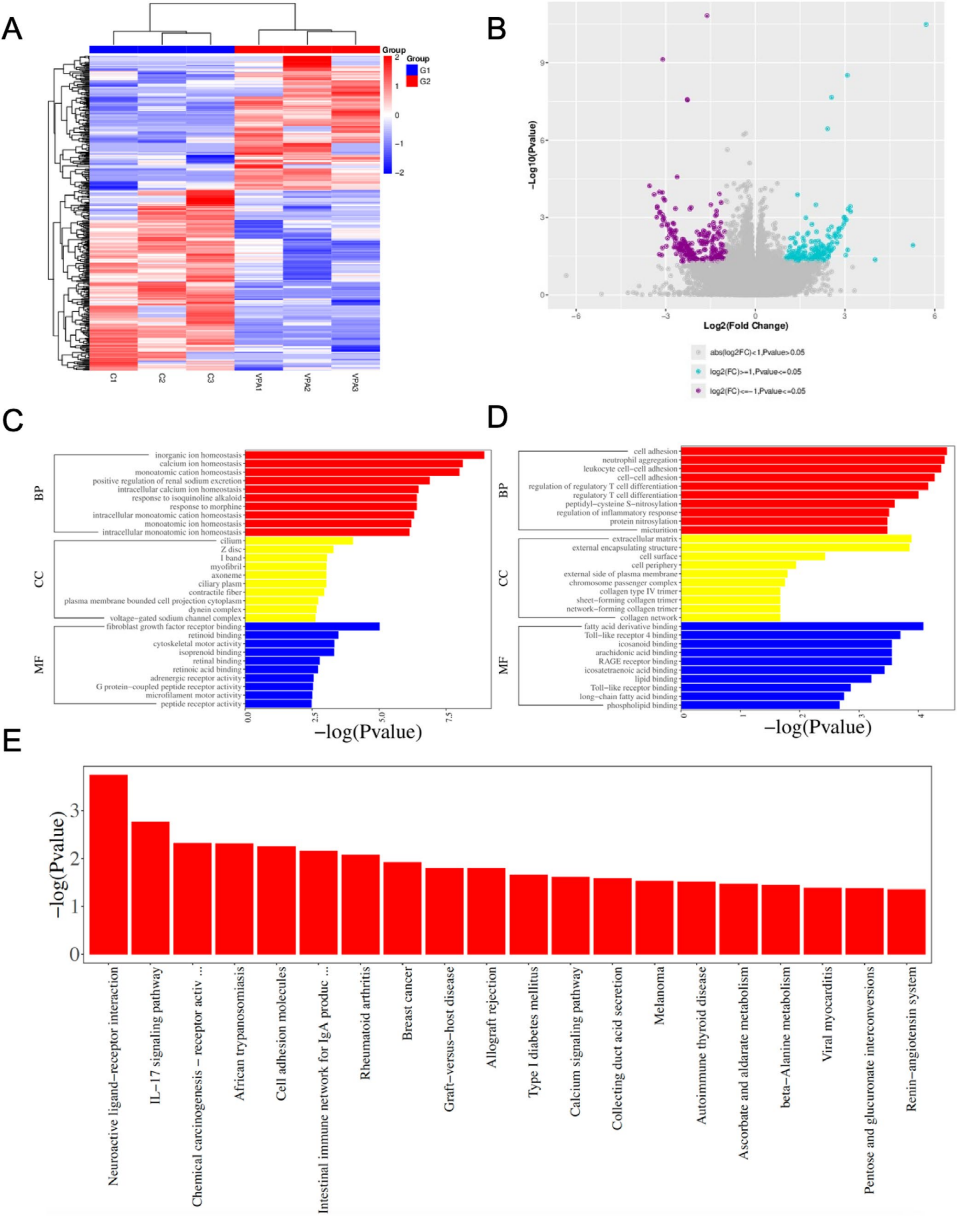
Valproic acid exposure altered gene expressions related with interneuron development. (A) Heatmap of DEGs. (B) Volcano plots of DEGs. (C, D) GO enrichment analysis of the biological processes based on the downregulated and upregulated DEGs. (E) KEGG analysis of the biological processes based on DEGs. Ctr: *N* = 3, VPA (exposed on E10.5, E12.5, and E14.5): *N* = 3.

RNA‐Seq analysis was performed to explore the underlying mechanisms driving abnormal SST neuron development after prenatal VPA exposure. The heatmap and volcano plots indicated that cortical gene expression changed due to prenatal VPA exposure (Figure 6A,B). GO term analysis showed that downregulated genes were mainly enriched in ion homeostasis, while upregulated genes were associated with cell adhesion (Figure 6C,D). KEGG analysis further proved that the altered genes were mainly enriched in the neuroactive ligand‐receptor interaction pathway (Figure 6E). The results suggested that VPA might influence SST neuron development through the regulation of ion homeostasis.

## 4 Discussion

In this study, we found that prenatal VPA exposure impaired the cognition of offspring. We also discovered that VPA exposure disrupted the migration and distribution of cortical interneurons in a time‐dependent manner.

Clinical studies demonstrated that prenatal VPA exposure impaired the children's cognition [29, 30, 31]. However, confounding factors such as seizures and medication usage in clinical practice interfere with the results. Epilepsy during pregnancy increased the probability of fetal growth restriction and neurodevelopmental disorders in offspring [32, 33]. To focus on the effects of VPA on neurodevelopment, we established a non‐teratogenic mouse model of maternal VPA exposure. We used 6‐ to‐week‐old pregnant C57BL/6 mice and injected VPA intraperitoneally after E10.5 to avoid causing malformations of offspring.

We started VPA injections after E10.5 when the development of major organs in mice was complete and neurogenesis began [34]. The body weight of the mice at P0 and P15, as well as the normal labor rate, was similar across groups, suggesting that VPA exposure did not cause major malformations in the offspring in the model we built.

In clinical practice, patients take VPA daily. Therefore, it remains unclear whether there is a critical time point during pregnancy when VPA exposure most significantly impairs neurodevelopment in offspring. A French cohort study divided pregnancy into three trimesters and found that VPA exposure in the first trimester was not associated with neurodevelopmental disorders in offspring, whereas continued exposure through the second and third trimesters significantly increased the probability of cognitive disorders [35]. This suggested that prenatal VPA exposure influenced offspring neurodevelopment in a time‐dependent manner. During the embryonic neurogenesis stage in mice, each day corresponds to 14–15 days of human development [36]. Therefore, we established a pregnant mouse model with a single VPA injection at E10.5, E12.5, or E14.5 to simulate exposure 1 month apart during human pregnancy. Our results indicated that VPA exposure at all three time points led to cognitive decline in offspring, with E12.5 being the most affected. At this stage, anxiety, long‐term memory loss, and working memory impairment were observed, suggesting that VPA had a time‐dependent effect on neurodevelopment.

Several studies reported that defects in interneuron development were linked to neurodevelopmental disorders [37, 38, 39]. We found that VPA exposure during pregnancy resulted in the abnormal distribution of cortical SST^+^ interneurons in offspring mice at P0. Previous studies also observed that prenatal VPA exposure at E12.5 and E13.5 caused abnormal positioning of neurons in the prefrontal and sensorimotor cortex of offspring mice [27], but the specific types of neurons were not identified. Our findings suggested that the abnormal migration of interneurons might be involved in this process. Aberrant interneuron migration was found in some psychiatric diseases [40, 41]. Abnormal cortical interneuron positioning was discovered in patients with 22q11 deletion syndrome, manifesting as schizophrenia and bipolar disorder [42].

We also found that the distribution of interneurons in offspring mice was impaired by intrauterine VPA exposure. Furthermore, the exposure primarily affected SST^+^ cells, indicating a subtype specificity among interneurons. Interneurons are highly heterogeneous and can be classified into various subtypes according to their morphological and electrophysiological properties. We speculated that the abnormal distribution of interneuron subtypes might be related to the disrupted differentiation process. Differentiation of interneurons could be influenced by external factors, such as hypoxia [43], nutrient deficiency [44] and infection [45]. Neonatal interneurons expressing SST were reduced when exposed to perinatal inflammation [46] and placental dysfunction [47]. Chronic stress also influenced interneuron differentiation by transcriptionally regulating DNA methylation [48].

There are several questions that warrant further study. The results suggest that the effects of VPA exposure are subtype selective, mainly affecting SST^+^ cells in the cortex and hippocampus. RNA‐Seq analysis showed that changes in ion homeostasis and cell adhesion might be the underlying mechanisms. The biological processes and pathways still need to be clarified. Moreover, the influence of prenatal VPA exposure in the context of epilepsy has yet to be investigated. A pregnant mouse model with epilepsy is in the plan. At the same time, the causal relationship between interneuron development and cognitive decline needs to be validated. The underlying molecular mechanisms also remain to be elucidated.

## 5 Conclusion

In conclusion, this study highlights the connection between cognitive impairment in offspring due to prenatal VPA exposure and abnormal interneuron development. The results suggest that interneurons could be a promising therapeutic target for addressing cognitive dysfunction caused by prenatal VPA exposure.

## Author Contributions

Jing Ding and Xin Wang contributed to the study design. Kaiyuan Shen, Yandong Zhang, and Yunyun Huang contributed to the experimentation. Kaiyuan Shen and Yunli Xie contributed to the data analysis. Kaiyuan Shen, Yunli Xie, Jing Ding, and Xin Wang contributed to the final manuscript preparation.

## Disclosure

The authors have nothing to report.

## Ethics Statement

All experimental procedures were performed in accordance with guidelines approved by the Animal Care and Use Committee of Fudan University.

## Conflicts of Interest

The authors declare no conflicts of interest. Part of the figures was created with http://BioRender.com.

## Data Availability

The data that support the findings of this study are available from the corresponding author upon reasonable request.
